# Beyond Analgesia: Preliminary Observations of Appetite and Weight Changes After Spinal Cord Stimulation

**DOI:** 10.7759/cureus.108037

**Published:** 2026-04-30

**Authors:** Rafael J Sanchez-Dopazo, Joseph E Mouhanna, Natasha Mehta, Rockeven B Desir

**Affiliations:** 1 Pain Medicine, Larkin Community Hospital, Miami, USA; 2 Pain Medicine, Larkin Community Hospital, Hialeah, USA; 3 Medicine, Medical University of the Americas, Charlestown, KNA

**Keywords:** appetite changes, appetite regulation, chronic pain management, implantable neurostimulation devices, neuromodulation, neuropathic pain, pain medicine outcomes, retrospective cohort study, spinal cord stimulation (scs), weight loss

## Abstract

Spinal cord stimulation (SCS) is an established therapy for chronic pain, primarily used for analgesia; however, its broader physiologic effects remain poorly characterized. This retrospective observational cohort study evaluated whether SCS implantation was associated with changes in appetite and body weight in patients with chronic pain. Eighteen patients with permanent SCS implants (10 females, eight males; aged 43-75 years) were reviewed. Outcomes included weight loss, weight gain, or no change in weight, along with self-reported appetite changes following implantation. Weight loss was observed in six patients (33%), ranging from 6.8 to 22.6 kgs, all of whom reported decreased appetite. Two patients (11%) experienced weight gain, while 10 patients (56%) demonstrated no significant weight change. Overall, seven patients (39%) reported decreased appetite after SCS implantation; however, decreased appetite did not consistently translate into measurable weight loss. These findings suggest that SCS may influence appetite regulation in a subset of patients with chronic pain, potentially through central neuromodulatory mechanisms beyond analgesia. Larger prospective studies with standardized metabolic and weight assessments are needed to further evaluate this relationship and its clinical implications.

## Introduction

Spinal cord stimulation (SCS) is an established neuromodulation therapy used in the treatment of chronic, refractory pain conditions, including neuropathic pain, failed back surgery syndrome (FBSS), and complex regional pain syndrome (CRPS). The therapy involves the delivery of electrical stimulation to the dorsal columns of the spinal cord through an implanted electrode system. This stimulation modulates nociceptive signaling pathways and reduces pain perception by altering neural activity in spinal and supraspinal pathways involved in pain transmission [1].

Over the past several decades, spinal cord stimulation has become an important therapeutic option for patients who do not respond adequately to conservative treatments such as pharmacologic therapy, physical rehabilitation, or interventional pain procedures [2]. Clinical guidelines and consensus recommendations support the use of spinal cord stimulation for appropriately selected patients with chronic neuropathic pain syndromes [3]. Evidence from randomized clinical trials has demonstrated that SCS can provide superior pain relief compared with conventional medical management, particularly in patients with failed back surgery syndrome [4].

In addition to improvements in pain intensity, several studies have reported enhancements in functional outcomes and quality of life among patients undergoing spinal cord stimulation therapy [4-6]. Systematic reviews and meta-analyses have further supported the effectiveness of SCS in chronic pain management, demonstrating reductions in pain scores and improved patient-reported outcomes [5-9]. Advances in stimulation technology, including high-frequency systems and improved programming capabilities, have expanded the therapeutic potential of SCS [10].

Although the primary therapeutic objective of spinal cord stimulation is pain reduction, neuromodulation therapies may influence physiologic processes beyond nociceptive signaling. Electrical stimulation of neural pathways can alter autonomic function, neurochemical signaling, and central nervous system activity through complex neural networks extending beyond the spinal cord [11].

Clinical observations suggest that some patients undergoing SCS implantation report changes in appetite and body weight following treatment. These observations raise the possibility that SCS may influence neural circuits involved in appetite regulation, potentially through modulation of autonomic pathways or interactions with central regulatory centers such as the hypothalamus [12]. However, this relationship remains poorly characterized in the literature [13]. The purpose of this study was to descriptively evaluate changes in appetite and body weight among patients undergoing spinal cord stimulator implantation for chronic pain.

## Materials and methods

This study consisted of a retrospective review of de-identified human patient data obtained from a pain management clinic. Patients had previously consented to the use of their clinical data for research purposes. All data were de-identified prior to analysis to ensure patient confidentiality.

A retrospective observational analysis was conducted to evaluate changes in appetite and body weight among patients who underwent permanent spinal cord stimulation implantation for chronic pain. This study represents a convenience sample of all eligible patients with available follow-up documentation; no formal sample size calculation was performed.

Medical records were reviewed for patients with documented follow-up after implantation. Demographic variables, including age and sex, were recorded. Baseline weight prior to implantation and follow-up weight measurements were obtained when available.

Patients were included if they were adults who underwent permanent SCS implantation and had documented follow-up data regarding both appetite and body weight. Follow-up was conducted within a six-month period following SCS implantation; however, the timing of assessments was not standardized due to the retrospective design, with variability in follow-up intervals among patients. Data were collected based on availability in the medical record. Patients without documented follow-up data were excluded. Weight outcomes were categorized into the following three groups: weight loss (any documented decrease in body weight), weight gain (any documented increase in body weight), and no change (the exact same documented weight at follow-up compared to baseline). Appetite outcomes were determined based on patient-reported documentation during follow-up visits.

To further explore potential differentiating characteristics within the cohort, a subgroup analysis was performed comparing patients who experienced weight loss with those who did not. Baseline demographic and clinical variables, including age, sex, and other available clinical characteristics, were compared descriptively between groups. Given the small sample size, formal multivariable adjustment was not performed; however, exploratory statistical comparisons were conducted using appropriate non-parametric and categorical tests where feasible. This analysis was intended to identify potential trends and generate hypotheses regarding patient-specific or procedural factors associated with differential responses to SCS. Potential confounding variables, including underlying pain diagnoses, medication use, and behavioral factors, were recognized; however, due to the small sample size and retrospective design, these variables were not formally controlled.

## Results

A total of 18 patients with permanent spinal cord stimulation implantation were included. The cohort consisted of 10 females and eight males, aged 43-75 years. Weight loss was observed in six patients (33%), with a range of approximately 6.8-22.7 kg. All six patients who experienced weight loss reported decreased appetite following SCS implantation. Two patients (11%) experienced weight gain after implantation. The remaining 10 patients (56%) demonstrated no measurable weight change, defined as identical recorded weight at follow-up compared to baseline. Overall, seven patients (39%) reported decreased appetite. Among these, six experienced weight loss, while one patient reported decreased appetite without measurable weight change. The distribution of weight change outcomes and appetite changes following SCS implantation is illustrated in Table 1.

**Table 1:** Subgroup comparison of clinical characteristics between patients with and without weight loss following spinal cord stimulation. Comparison of demographic and clinical variables between patients who experienced weight loss (n = 6) and those who did not (n = 12) after spinal cord stimulation (SCS) implantation. Variables include age, sex distribution, baseline body weight, weight change, and reported appetite changes. Patients in the weight-loss group demonstrated a trend toward higher baseline body weight and consistently reported decreased appetite. No significant differences were observed in age or sex distribution between groups. Weight change and decreased appetite showed the largest differences between groups, although findings should be interpreted as exploratory due to the small sample size and retrospective study design.

Variables	Weight loss (n=6)	No weight loss (n=12)	Effect size	95% CI	p-Value
Age (years), mean±SD	57.8±8.9	60.4±10.7	Δ = -2.6	-13.4 to 8.2	0.63
Sex: Female, n (%)	3 (50%)	7 (58%)	OR = 0.73	0.10 to 5.29	1.00
Sex: Male, n (%)	3 (50%)	5 (42%)	OR = 1.36	0.19 to 9.52	1.00
Baseline weight (kg), mean±SD	101.2±15.1	88.9±13.5	Δ = +12.3	-2.4 to 27.0	0.09
Weight change (kg), mean ± SD	-13.4±4.8	+0.8±2.6	Δ = -14.2	-19.1 to -9.3	<0.001
Decreased appetite, n (%)	6 (100%)	1 (8%)	OR = 96.5	3.8 to 2445.0	0.003

To further evaluate potential differentiating characteristics, a subgroup comparison was performed between patients who experienced weight loss (n = 6) and those who did not (n = 12). Patients in the weight loss group demonstrated a trend toward higher baseline body weight and more consistent reporting of appetite suppression following implantation. No clear differences were observed in age or sex distribution between groups. Due to the limited sample size, these findings did not reach statistical significance; however, the observed patterns suggest a possible phenotype of patients more susceptible to appetite modulation and subsequent weight loss following SCS therapy.

These findings demonstrate variability in physiologic responses following spinal cord stimulation therapy. While decreased appetite was observed in a subset of patients, it did not consistently translate into measurable weight change across all individuals. The subgroup trends further suggest that weight loss may be associated with specific patient-level characteristics, though these observations remain exploratory.

Subgroup analysis

**Figure 1:**
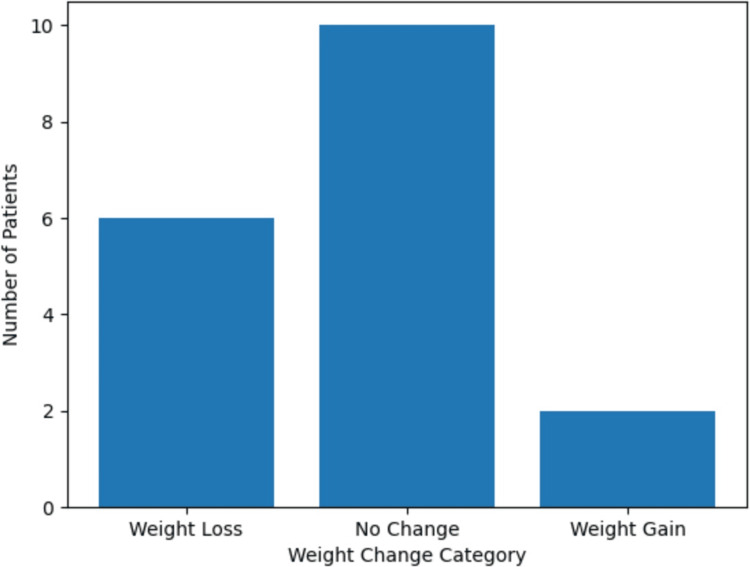
Post-spinal cord stimulation weight change and appetite outcomes in chronic pain patients. Distribution of weight change outcomes among patients following spinal cord stimulator (SCS) implantation (n = 18). Weight loss was observed in six patients (33%), weight gain in two patients (11%), and no weight change in 10 patients (56%). Additionally, seven patients (39%) reported decreased appetite following SCS implantation. The bar graph illustrates the distribution of patients across weight change categories after neuromodulation therapy.

A subgroup analysis was performed to compare patients who experienced weight loss following spinal cord stimulation (SCS) implantation (n = 6) with those who did not (n = 12). Comparative results are summarized in Figure 1. The mean age of patients in the weight-loss group was 57.8 ± 8.9 years, compared to 60.4 ± 10.7 years in the no-weight-loss group (Δ = -2.6 years, 95% CI: -13.4 to 8.2; p = 0.63). Sex distribution was similar between groups, with females comprising 50% (3/6) of the weight-loss group and 58% (7/12) of the no-weight-loss group (OR = 0.73, 95% CI: 0.10 to 5.29; p = 1.0).

Patients who experienced weight loss demonstrated a higher mean baseline body weight compared to those without weight loss (101.2 ± 15.1 kg vs. 88.9 ± 13.5 kg), with a mean difference of 12.3 kg (95% CI: -2.4 to 27.0; p = 0.09), indicating a trend toward higher baseline weight in this subgroup. As expected, the weight-loss group demonstrated a significantly greater reduction in body weight (-13.4 ± 4.8 kg) compared to the no-weight-loss group (+0.8 ± 2.6 kg), with a mean difference of -14.2 kg (95% CI: -19.1 to -9.3; p<0.001). Decreased appetite was reported in all patients in the weight-loss group (100%) compared to 8% (1/12) in the no-weight-loss group, corresponding to an odds ratio of 96.5 (95% CI: 3.8 to 2445.0; p = 0.003).

Overall, no statistically significant differences were observed in demographic variables between groups. However, the consistent reporting of decreased appetite among patients who experienced weight loss and the trend toward higher baseline body weight suggest the presence of a potential responder phenotype. Given the small sample size and retrospective design, these findings should be interpreted as exploratory and hypothesis-generating.

## Discussion

Spinal cord stimulation is a well-established therapy for chronic neuropathic pain, with substantial evidence supporting its effectiveness in reducing pain and improving functional outcomes [5,6,9]. While its primary mechanism involves modulation of nociceptive pathways within the dorsal columns, emerging evidence suggests that neuromodulation may influence broader neural networks, including autonomic and central regulatory systems [3,11,13].

In this study, a subset of patients reported decreased appetite following SCS implantation, with several experiencing clinically meaningful weight loss. These findings raise the possibility that SCS may influence physiologic processes related to appetite regulation. However, the relationship between neuromodulation and metabolic outcomes remains incompletely understood.

Appetite regulation is governed by complex interactions between peripheral metabolic signals and central neural pathways, particularly within hypothalamic and autonomic circuits. Although SCS primarily targets spinal pathways, it is plausible that electrical stimulation may indirectly influence these central regulatory systems. Despite these observations, decreased appetite did not uniformly result in weight loss, suggesting that additional factors, such as baseline metabolic status, medication use, dietary behavior, and physical activity, may influence outcomes.

This study has several important limitations. The very small sample size, retrospective design, lack of standardized follow-up intervals, reliance on patient-reported appetite changes, and absence of a control group significantly limit the generalizability of the findings. Additionally, no formal statistical analysis or correlation assessment was performed, and potential confounding variables could not be controlled.

Importantly, subgroup analysis revealed that patients who experienced weight loss demonstrated consistent reporting of appetite suppression and a trend toward higher baseline body weight compared to those without weight loss. While no statistically significant differences were identified, likely due to the small sample size, these patterns suggest the presence of a potential responder phenotype. This raises the possibility that certain patient-specific factors may predispose individuals to metabolic or appetite-related effects following SCS therapy. These limitations underscore that the findings should be interpreted as preliminary and hypothesis-generating rather than confirmatory. Future research should include larger retrospective or prospective cohorts, standardized follow-up intervals, objective metabolic measurements, and control groups to better evaluate the potential relationship between spinal cord stimulation and appetite regulation.

## Conclusions

In this retrospective observational cohort, a subset of patients undergoing spinal cord stimulator implantation reported decreased appetite, with several demonstrating associated weight loss. Although most patients did not experience measurable weight change, these findings raise the possibility that spinal cord stimulation may influence physiologic processes beyond pain modulation, including pathways involved in appetite regulation.

The observed variability suggests that appetite changes do not consistently translate into weight loss and are likely influenced by additional factors such as metabolic status, medication use, and behavioral patterns. Given the very small sample size, retrospective design, lack of standardized follow-up, and absence of a control group or statistical analysis, these findings should be interpreted as exploratory and hypothesis-generating. Further research with larger, prospective cohorts, standardized assessments, and objective metabolic measures is needed to better define the relationship between spinal cord stimulation and appetite regulation and to clarify its potential clinical implications.
